# Tailored Web-Based Smoking Interventions and Reduced Attrition: Systematic Review and Meta-Analysis

**DOI:** 10.2196/16255

**Published:** 2020-10-19

**Authors:** Amika Shah, Michael Chaiton, Dolly Baliunas, Robert Schwartz

**Affiliations:** 1 Dalla Lana School of Public Health University of Toronto Toronto, ON Canada; 2 Ontario Tobacco Research Unit Dalla Lana School of Public Health University of Toronto Toronto, ON Canada; 3 Office of Education Centre for Addiction and Mental Health Toronto, ON Canada

**Keywords:** internet, world wide web, smoking cessation, web-based intervention

## Abstract

**Background:**

The increasing number of internet users presents an opportunity to deliver health interventions to large populations. Despite their potential, many web-based interventions, including those for smoking cessation, face high rates of attrition. Further consideration of how intervention features impact attrition is needed.

**Objective:**

The aim of this systematic review is to investigate whether tailored web-based smoking cessation interventions for smokers are associated with reduced rates of attrition compared with active or passive untailored web-based interventions. The outcomes of interest were dropout attrition at 1-, 3-, 6-, and 12-month follow-ups.

**Methods:**

Literature searches were conducted in May 2018 and updated in May 2020 on MEDLINE (Medical Literature Analysis and Retrieval System Online), PsycINFO (Psychological Information), EMBASE (Excerpta Medica dataBASE), CINAHL (Cumulated Index to Nursing and Allied Health Literature), Scopus, and the Cochrane Tobacco Addiction Group Specialized Register with the following search terms: smoking cessation, tailored, or web- or internet-based. Included studies were published in English before or in May 2020 using a randomized controlled trial design. Studies were restricted to those with web-based delivery, a tailored intervention group, an untailored control group, and a reported outcome of smoking cessation. Studies were assessed for methodological quality using the Cochrane Risk of Bias tool. Two reviewers independently extracted the study characteristics and the number of participants lost to follow-up for each treatment group.

**Results:**

A total of 13 studies were included in the systematic review, of which 11 (85%) were included in the meta-analysis. Tailoring had no statistically significant effect on dropout attrition at 1-month (risk ratio [RR]=1.02, 95% CI 0.95-1.09; *P*=.58; *I*^2^=78%), 3-month (RR=0.99, 95% CI 0.95-1.04; *P*=.80; *I*^2^=73%), 6-month (RR=1.00, 95% CI 0.95-1.05; *P*=.90; *I*^2^=43%), or 12-month (RR=0.97, 95% CI 0.92-1.02; *P*=.26; *I*^2^=28%) follow-ups. Subgroup analyses suggested that there was a statistically significant effect of tailoring between the active and passive subgroups at 1-month (*P*=.03), 3-month (*P*<.001), and 6-month *(P*=.02) follow-ups but not at 12-month follow-up (*P*=.25).

**Conclusions:**

The results suggest that tailoring of web-based smoking cessation interventions may not be associated with reduced rates of dropout attrition at 1-, 3-, 6-, or 12-month follow-ups. Significant differences between studies that include untailored active and passive control groups suggest that the role of tailoring may be more prominent when studies include a passive control group. These findings may be because of variability in the presence of additional features, the definition of smokers used, and the duration of smoking abstinence measured. Future studies should incorporate active web-based controls, compare the impact of different tailoring strategies, and include populations outside of the Western countries.

## Introduction

### Background

Smoking is the leading cause of preventable death [1]. Annually, over 7 million deaths worldwide are attributed to cigarette smoking [2], leading to a global loss of 150 million disability-adjusted life years [3]. The majority of smokers want to quit [4]; however, it can take 30 or more attempts to successfully quit [5]. Although smoking cessation is difficult, the price of not quitting is high—over 50% of long-term smokers die from smoking [6,7]. Fortunately, evidence-based smoking cessation interventions can double or triple the chances that a quit attempt will result in long-term cessation [8]. However, most effective clinical interventions have been found to be more costly and to have lower reach compared with public health interventions [9].

### Web-Based Interventions

Interventions delivered over the internet, termed *web-based interventions*, have the potential to increase the public health impact of smoking cessation interventions because of their widespread use and scalability [10]. Despite their potential, few web-based interventions succeed in delivering the full treatment intended because of high rates of attrition. In a seminal paper, Eysenbach [11] delineated 2 types of attrition in web-based interventions: nonusage and dropout. The former refers to users who do not use the web-based intervention, whereas the latter refers to users who do not complete the follow-up study procedures. Eysenbach [11] hypothesized that both types of attrition are related and may be explained by a common experience of the user *losing interest*. As such, research that contributes to the *science of attrition* is recommended to better understand the phenomenon of attrition in the context of trials of web-based interventions [11].

Like many web-based interventions [11], web-based smoking cessation interventions often report high rates of dropout attrition [12], posing threats to the validity of evidence surrounding these interventions. Although studies on smoking cessation often employ intention-to-treat analyses, where those lost to follow-up are assumed to be smokers, high rates of dropout attrition increase the risk of attrition bias, which may lead to the underestimation of the effectiveness of web-based smoking cessation interventions. Such findings have been reported by systematic reviews of web-based smoking cessation interventions that have identified attrition bias as a critical challenge in assessing the effectiveness of these interventions [12-14]. In addition, bias caused by attrition makes it difficult to identify features of web-based smoking cessation interventions that are most effective in promoting smoking cessation. Thus, further consideration of the features that influence dropout attrition is needed to fully understand the impact of web-based smoking cessation interventions, the features that characterize effective interventions, and their mechanisms of action.

### Tailoring

Tailoring is one feature that has received significant interest [13]. Tailored print smoking cessation materials have been found to be more effective than untailored materials [15,16], suggesting that this feature may be important for their web-based equivalents. Likewise, systematic reviews of web-based smoking cessation interventions have highlighted the importance of tailoring in promoting smoking cessation [12-14]. For instance, Taylor [12] investigated the effectiveness of interactive, tailored, and combined (tailored and interactive) web-based interventions compared with nonactive and active control interventions. Combined interactive and tailored interventions were found to be moderately more effective than nonactive controls (eg, printed self-help guide) but not more effective than active controls (eg, counseling sessions) [12]. The authors noted that many of the studies had high rates of attrition, making it difficult to assess the effectiveness of tailored web-based smoking cessation interventions [12]. Thus, the ongoing challenge of high dropout attrition presents a need to explore the effect of intervention design features, such as tailoring, on attrition [15,17].

### Objectives

This study aims to investigate across randomized controlled trials (RCTs), whether tailored active web-based smoking cessation interventions reduce dropout attrition at 1-, 3-, 6-, and 12-month follow-ups compared with an untailored active or passive control. In the context of this review, we define the terms *tailoring*, *active*, and *passive* as follows.

#### Tailoring

The term *tailoring* is often used interchangeably with *personalization* and *targeting*; however, important differences exist among these 3 terms [17]. Personalization refers to materials that have been customized using an individual’s name, whereas targeting involves designing materials for a particular subgroup or population with one or more shared characteristics (eg, youth) [17]. In contrast, tailored materials refer to materials that are designed for the characteristics of a particular individual based on individual assessment (eg, quit date, level of motivation, and self-efficacy) [17]. These terms may be viewed as existing on a continuum ranging from generic to individually tailored [17], with each term varying in the level in which they consider and incorporate characteristics of the user into the intervention. Although we recognize the differences among these terms, for simplicity, we operationalize the term *tailored* as an umbrella term that encompasses personalization, targeting, and tailoring to the individual.

#### Active

The term active is defined as interventions or control groups that require more than one engagement by the user. The multiple engagements must be part of the intervention and not simply part of the study procedures. For instance, a study with a single tailored email that requires multiple follow-up procedures would not be considered active in the context of this review. The authors chose to focus on active interventions, as attrition is more likely to be an issue for interventions and studies that require multiple engagements over time, rather than a single engagement.

#### Passive

The term passive is defined as control groups that require a single engagement by the user. The authors chose to compare studies with either an active or passive control group to assess whether the effect of tailoring differs between studies that compare active tailored interventions to control groups that require similar (active) or different (passive) levels of engagement.

## Methods

### Protocol and Registration

This review is designed and reported in line with the PRISMA (Preferred Reporting Items for Systematic reviews and Meta-Analyses) reporting guidelines (for checklist, see Multimedia Appendix 1) [18]. A protocol was developed to guide this review; however, the protocol was not registered. Details outlined in the protocol included the study rationale, research question, eligibility criteria, study selection process, outcomes of interest, and the processes followed in conducting the meta-analysis. No deviations were made from the protocol from start to the final review [18].

### Data Source and Search Strategy

Searches were conducted using the following electronic databases: MEDLINE (Medical Literature Analysis and Retrieval System Online), PsycINFO (Psychological Information), EMBASE (Excerpta Medica dataBASE), CINAHL (Cumulated Index to Nursing and Allied Health Literature), Scopus, and the Cochrane Tobacco Addiction Group Specialized Register. Search strategies for each database were created using synonyms of the 4 main search concepts: (1) *smoking cessation* (outcome measure), (2) *tailoring* (intervention feature), (3) *web-* or *internet-based* (technology), and (4) *randomized control trial* (study design). A sample search strategy for MEDLINE is provided in Multimedia Appendix 2. An initial search was conducted in May 2018 for studies published in or before May 2018. The review was later updated in May 2020 to identify any studies published between May 2018 and May 2020. Search results were exported and managed on EndNote Web.

### Study Selection

**Figure 1:**
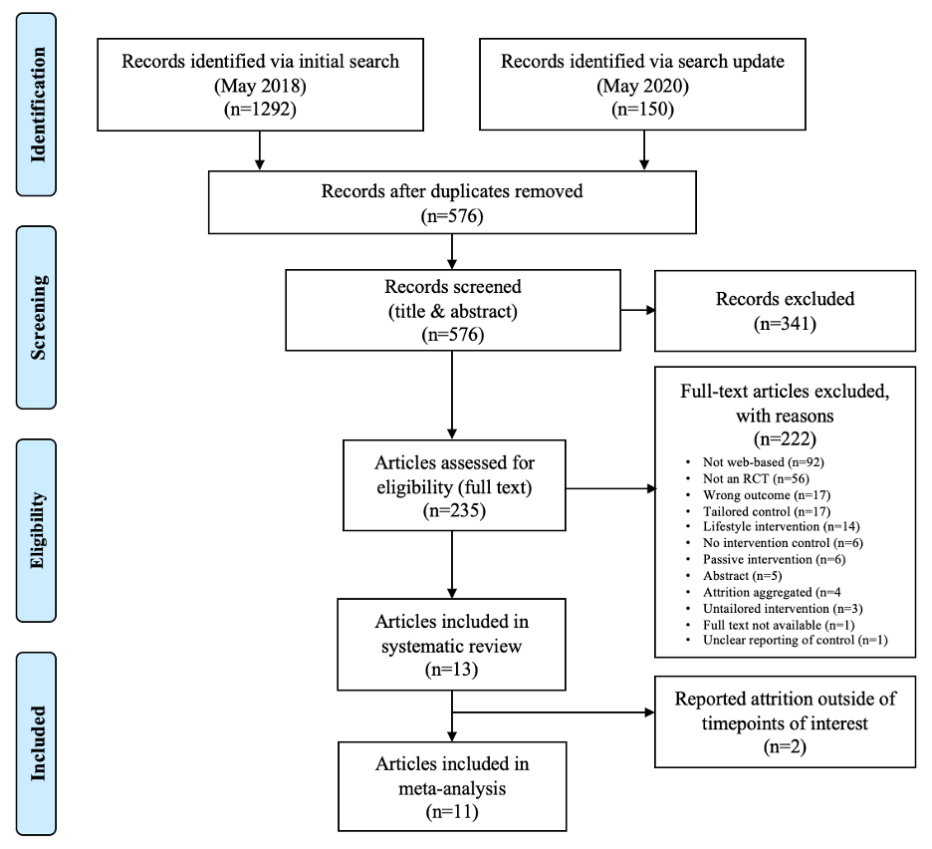
PRISMA (Preferred Reporting Items for Systematic reviews and Meta-Analyses) diagram.

Two authors (AS and MC) independently reviewed papers retrieved from the search strategy by title and abstract. Rayyan, a web-based application for systematic reviews, was used to facilitate the screening process between the 2 reviewers. The inclusion criteria were that studies (1) employed any type of randomized controlled trial (RCT) design (eg, crossover trials, parallel trials, and factorial trials), (2) included a tailored web-based smoking cessation intervention, (3) had an untailored control intervention (both web-based or nonweb-based were accepted), and (4) assessed smoking cessation through any method (ie, point prevalence estimate, self-report, and biochemical validation) at least 1 month after the start of the intervention. Papers could be published in or before May 2020. Only peer-reviewed articles published in English with a study population of smokers were included in the analysis. Dissertations, poster abstracts, and studies that described lifestyle interventions (interventions that targeted multiple health behaviors) were excluded (Figure 1).

Studies were selected based on the tailored intervention component. If the tailored component was not delivered online (ie, a computer-tailored letter that was printed and mailed to the participant), the study was excluded. Multicomponent interventions were included; however, if the tailored component was not delivered on the web (eg, tailored text messages accompanied by a web page), the study was deemed ineligible. These criteria were used to isolate the impact of tailoring and increase the comparability of the interventions included in the meta-analysis.

Studies that met the exclusion criteria when reviewed by title and abstract were excluded. The remaining studies were moved on to the next stage, where they were assessed using the full text. Full-text assessments of studies were later conducted for eligibility, with reasons for exclusion recorded. Disagreements in study eligibility were resolved through discussions between the two reviewers (AS and MC).

### Study Quality

**Figure 2:**
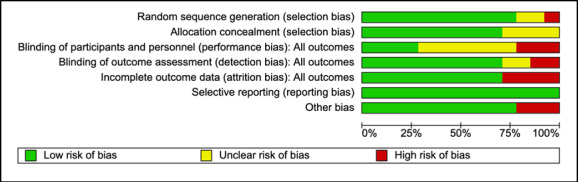
Risk of bias graph.

Studies were assessed for methodological quality using the Cochrane Risk of Bias Tool [19], with reasons for risk level recorded (Figure 2). Risk of bias assessment was conducted at the study level. Risk of bias assessments were considered during the data interpretation stage.

### Data Extraction and Analysis

The following details were extracted from the included studies: author, year of publication, study location, sample, population characteristics, intervention, theoretical framework used, tailored components, control group used, follow-up period, and key findings. In addition, the reported number of individuals lost to follow-up for the intervention and control arms were extracted from each study and compared against the original sample of individuals allocated to the intervention and control arms. If missing data were related to the outcome of interest (dropout attrition), the authors were contacted for additional information. Risk ratios (RRs) and 95% CIs were used to measure the difference in attrition between tailored interventions and untailored control groups. All significance tests were 2-tailed, with *P*<.05 considered statistically significant. A meta-analysis was conducted with the software RevMan 5 (Cochrane Organization) using the Mantel–Haenszel fixed effects model to pool the RRs. A fixed effects model was chosen as the most conservative model with the expectation that there would be some heterogeneity in design. The *I*^2^ statistic was used to assess statistical heterogeneity at 3 upper limits (low, *I*^2^<25%; moderate, *I*^2^ between 25% and 75%; high, *I*^2^>75%) [20]. A sensitivity analysis was conducted to examine the effect of potential outliers on the meta-analysis results.

## Results

### Search Results

The results of the search strategy are summarized in the PRISMA flowchart shown in Figure 1. When the first search was conducted in May 2018, the search strategy yielded a total of 1292 studies, of which 801 (62.0%) were duplicates. Upon screening the remaining 491 studies by title and abstract, 274 (55.8%) studies were excluded. The remaining 217 studies were later screened by the full text, and 204 (94.0%) studies were excluded for not meeting the inclusion criteria. From this initial search, 13 studies were included in the systematic review [21-33]. The search update conducted in May 2020 identified a total of 150 studies. After removing duplicates, 56.7% (85/150) of the studies were screened by title and abstract against the inclusion criteria, and 79% (67/85) studies were subsequently excluded. The remaining 21% (18/85) of the studies were screened by their full texts. Within these 18 studies, 2 (11%) appeared to meet the inclusion criteria, although neither study could be included in the analysis. At the time of the search update, the full text of Kahler [34] was not available, and thus, the details required for the meta-analysis could not be extracted. Additionally, due to unclear details regarding the control intervention, we excluded the study by Altendorf et al [35].

### Study Characteristics

Details of the included studies, such as the author, year of publication, study location, sample, population characteristics, intervention, theoretical framework used, tailored components, control, and key findings, are outlined in Multimedia Appendix 3. The included studies were published from 2008 to 2018 in the following countries: the United States [21,24,26,27,32,33], Switzerland [22], Norway [23], Australia [25], France [28], Spain [29], Denmark [30], and the Netherlands [31]. Of the 13 included studies, 4 (31%) studies used an active control intervention, whereas 9 (69%) studies used a passive control intervention.

The 14 trials included a total of 12,661 participants: 6538 (51.64%) randomized to a tailored intervention and 6123 (48.63%) to an untailored control intervention. Participants were recruited from a variety of settings, including postsecondary institutions [24,29], hospitals or clinics [26,27,31,32], a Quitline [24], and from a website [21,22,28,33]. Follow-up periods ranged from 1 to 18 months. Five studies reported follow-up at 1 month [23,25,30,32,33], 6 studies at 3 months [21-23,26,28,33], 9 studies at 6 months [21,22,26-31,33], 6 studies at 12 months [21,22,26-31,33], and 1 study at 18 months [21]. One study reported outcomes at 8, 20, and 30 weeks [26] and another at 7 months [27]. Of the 13 studies included in the systematic review, 7 (54%) studies [23-26,28,29,33] reported significant differences in smoking cessation between the tailored intervention and untailored control at one or more follow-up periods.

### Study Quality

Most studies included in the analysis were found to have an unclear or high risk of performance bias (Figure 2). Moreover, 4 studies were found to be at high risk of attrition bias as characterized by studies with more than 50% of the sample randomized to a condition lost to attrition.

### Attrition

All the 13 included studies reported dropout attrition, whereas only 1 (8%) study [25] reported nonusage attrition. Dropout attrition across the 13 studies ranged from 5% to 67% for tailored interventions and from 3% to 64% for untailored interventions (Table 1). Among the 13 studies that met the inclusion criteria, 2 (15%) studies [24,25] were excluded from the meta-analysis, as they reported dropout attrition at timepoints that could not be compared with any other study included in the analysis. Although Borland [25] included follow-ups at 1 and 7 months, attrition was not reported for the 1-month follow-up, and thus, this study was excluded from the meta-analysis. The exclusion of these 2 studies resulted in a total of 11 studies that were included in the meta-analysis. The results of the meta-analysis with respect to the follow-up periods of interest (1, 3, 6, and 12 months) are described in the following section.

**Table 1:** Summary of attrition (n=13).

1st author, reference	Attrition type reported	Follow-up period	Intervention sample, n	Intervention loss to follow-up, n (%)	Control sample, n	Control loss to follow-up, n (%)
**An [24]**						
	Dropout	8 weeks	257	13 (5.1)	260	8 (3.1)
	Dropout	20 weeks	257	24 (9.3)	260	20 (7.7)
	Dropout	30 weeks	257	23 (8.9)	260	30 (11.5)
**Borland [25]**						
	Dropout and nonusage	1 month	809	Not reported	422	Not reported
	Dropout and nonusage	7 months	809	104 (12.9)	422	66 (15.6)
**Das [26]**						
	Dropout	3 months	105	16 (15.2)	111	20 (18.0)
	Dropout	6 months	105	13 (12.4)	111	15 (13.5)
	Dropout	12 months	105	13 (12.4)	111	14 (12.6)
**Graham [21]**						
	Dropout	3 months	651	151 (23.2)	679	142 (20.9)
	Dropout	6 months	651	168 (25.8)	679	154 (22.7)
	Dropout	12 months	651	180 (27.7)	679	187 (27.5)
	Dropout	18 months	651	201 (30.9)	679	213 (31.4)
**Harrington [27]**	Dropout	6 months	748	98 (13.1)	740	89 (12.0)
**Mavrot [22]**						
	Dropout	3 months	580	290 (50.0)	580	251 (43.3)
	Dropout	6 months	580	353 (60.9)	580	331 (57.1)
**Nyguyen [28]**						
	Dropout	3 months	1242	639 (51.4)	1236	720 (58.3)
	Dropout	6 months	1242	667 (53.7)	1236	729 (59.0)
	Dropout	12 months	1242	732 (58.9)	1236	793 (64.2)
**Pardavila-Belio [29]**	Dropout	6 months	133	19 (14.3)	122	11 (9.0)
**Skov-Ettrup [30]**						
	Dropout	1 month	453	106 (23.4)	452	64 (14.2)
	Dropout	6 months	453	67 (14.8)	452	75 (16.6)
	Dropout	12 months	453	84 (18.5)	452	66 (14.6)
**Smit [31]**						
	Dropout	6 months	132	89 (67.4)	119	74 (62.2)
	Dropout	12 months	132	57 (43.2)	119	55 (46.2)
**Tsoh [32]**						
	Dropout	1 month	23	5 (21.7)	19	5 (26.3)
	Dropout	2 months	23	9 (39.1)	19	6 (31.6)
**Wangberg [23]**						
	Dropout	1 month	1029	613 (59.6)	1043	640 (64.1)
	Dropout	3 months	1029	648 (63.0)	1043	644 (61.7)
	Dropout	12 months	1029	303 (29.4)	1043	300 (28.8)
**Westmaas [33]**						
	Dropout	1 month	376	98 (26.1)	340	95 (27.9)
	Dropout	3 months	376	134 (35.6)	340	112 (32.9)
	Dropout	6 months	376	156 (41.5)	340	136 (40.0)

#### One-Month Follow-Up

Of the 11 studies, 6 (55%) studies were included in the meta-analysis and 4 (36%) studies [23,30,32,33] reported attrition at 1-month follow-up. Only 1 (9%) study [23] compared a tailored active intervention with an untailored active control. There were no significant differences in the risk of attrition between tailored interventions and untailored controls at 1-month follow-up (RR=0.97, 95% CI 0.91-1.04; *P*=.41). Across the 3 studies [30,32,33] that compared a tailored active intervention with an untailored passive control, the risk of attrition was higher among tailored interventions at 1-month follow-up (RR=1.20, 95% CI 1.00-1.44; *P*=.04). This estimate was statistically significant, although it had high heterogeneity (*I*^2^=79%). The test for subgroup differences indicated significant differences between studies that included an active control and those that included a passive control at 1-month follow-up (χ^2^_1_=4.7; *P*=.03).

**Figure 3:**
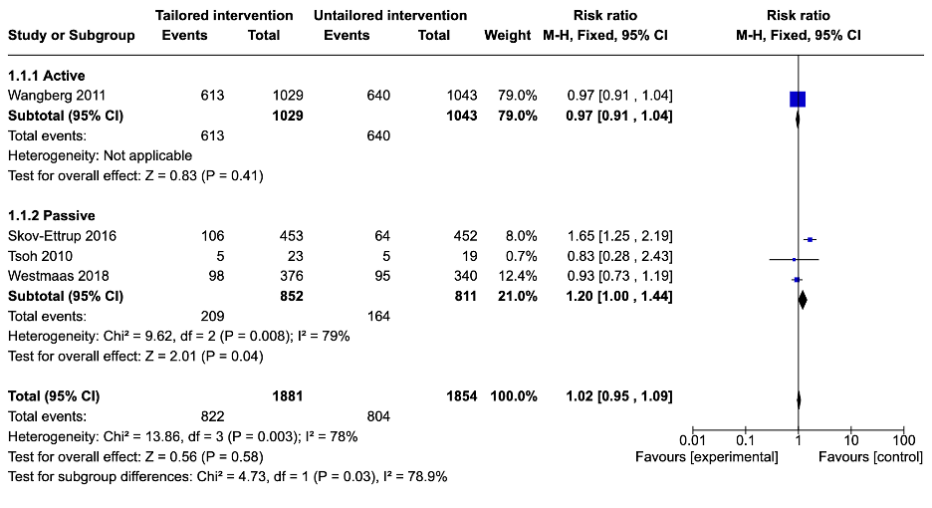
Comparison of attrition between tailored and untailored conditions at 1-month follow-up by control type.

When the 4 studies [23,30,32,33] were pooled, no significant differences in the risk of attrition between tailored interventions and untailored controls were found at 1-month follow-up (RR=1.02, 95% CI 0.95-1.09; *P*=.58) with high heterogeneity (*I*^2^=78%) in the estimate (Figure 3). These findings suggest that tailoring had no effect on dropout attrition at 1-month follow-up.

#### Three-Month Follow-Up

Of the 11 studies included in the meta-analysis, 6 (55%) reported attrition at 3-month follow-up [21-23,26,28,33]. A total of 27% (3/11) of the studies [21-23] compared a tailored active intervention with an untailored active control. When these 3 studies were pooled, tailored interventions were at a significantly higher risk of attrition compared with untailored controls (RR=1.06, 95% CI 1.00-1.13; *P*=.03) with moderate heterogeneity in the estimate (*I*^2^=42%). The remaining 3 studies [26,28,33] compared a tailored active intervention with an untailored passive control. Among these studies, the risk of attrition was lower for the tailored intervention than for the untailored intervention (RR=0.91, 95% CI 0.85-0.97; *P*=.006). This estimate was statistically significant with moderate heterogeneity (*I*^2^=43%). Significant differences between the active and passive subgroups were found at 3-month follow-up (χ^2^_1_ =11.9; *P*<.001).

**Figure 4:**
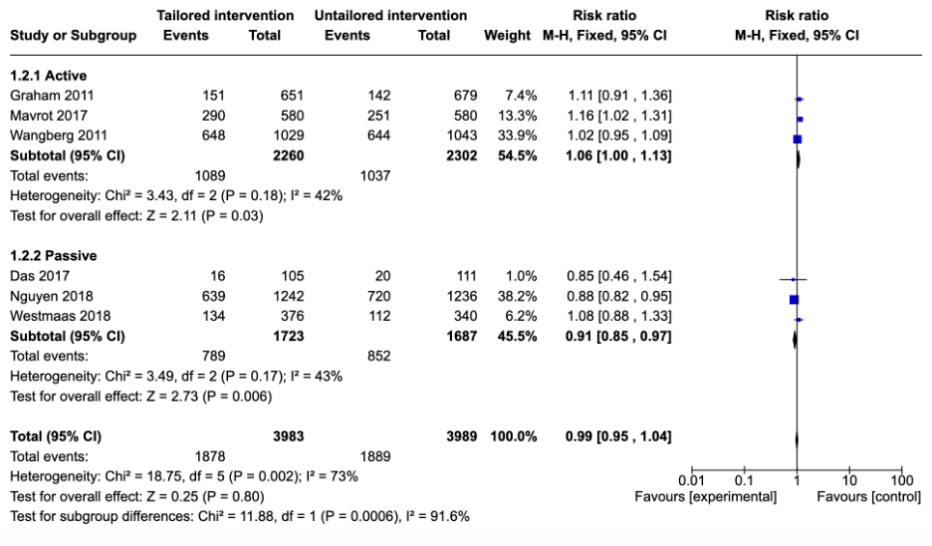
Comparison of attrition between tailored and untailored conditions at 3-month follow-up by control type.

When the 6 studies [23-25,28,30,35] that reported attrition at 3-month follow-up were pooled (Figure 4), no significant differences were found in the risk of attrition between the tailored interventions and their untailored controls (RR=0.99, 95% CI 0.95-1.04; *P*=.80) with high heterogeneity in the estimate (*I*^2^=73%). This finding suggests that tailoring had no effect on dropout attrition at 3-month follow-up.

#### Six-Month Follow-Up

Of the 11 studies included in the meta-analysis, 9 (82%) reported attrition at 6-month follow-up [21,22,26-31,33]. Of these 9 studies, 2 compared a tailored active intervention with an untailored active control [21,22]. Differences in the risk of attrition between the tailored and untailored groups for these 2 studies were not significant (RR=1.09, 95% CI 1.00-1.19; *P*=.06) with no heterogeneity in the estimate (*I*^2^=0%). In addition, 7 of the 9 studies (78%) [26-31,33] compared a tailored active intervention with an untailored passive control. Among these 7 studies, there were no significant differences in the risk of attrition between the tailored intervention relative to the untailored control (RR=0.96, 95% CI 0.90-1.02; *P*=.16) with low heterogeneity in the estimate (*I*^2^=22%). The test for subgroup differences suggested that significant differences existed between the active and passive subgroups at 6-month follow-up (χ^2^_1_=5.4; *P*=.02).

**Figure 5:**
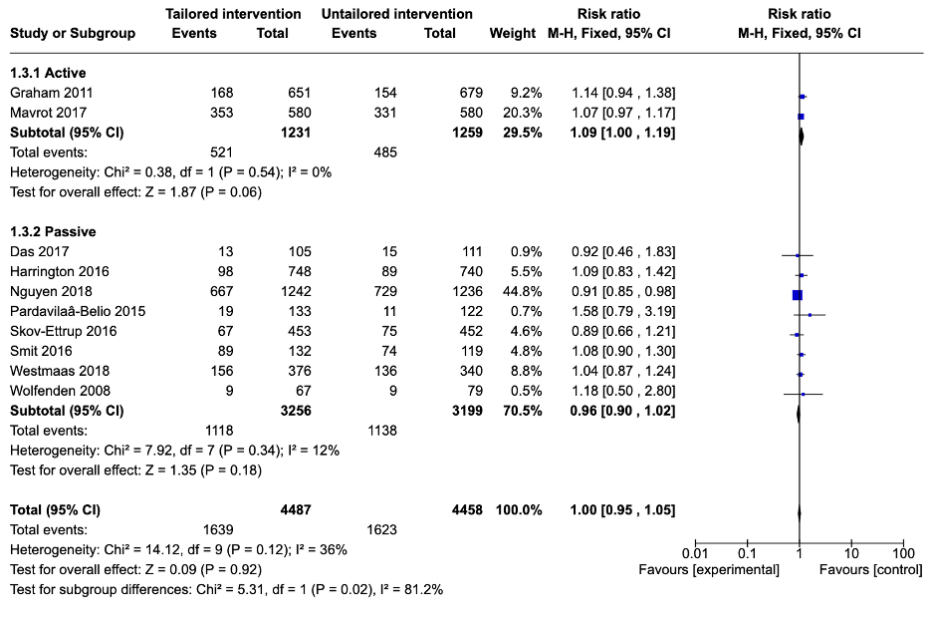
Comparison of attrition between tailored and untailored conditions at 6-month follow-up by control type.

When the 9 studies [21,22,26-31,33] that reported attrition at 6-month follow-up were pooled, no differences were found in dropout attrition between the tailored interventions and untailored controls (RR=1.00, 95% CI 0.95-1.05; *P*=.90) with moderate heterogeneity (*I*^2^=43%, Figure 5). This suggests that tailoring had no effect on the risk of attrition at 6-month follow-up.

#### Twelve-Month Follow-Up

Of the 11 studies included in the meta-analysis, 6 (55%) reported attrition at 12-month follow-up [21,23,26,28,30,31]. Of these 6 studies, 2 studies [21,23] compared a tailored active intervention with an untailored active control. The pooled effect of these 2 studies found that there were no significant differences in the risk of attrition between tailored interventions and untailored controls (RR=1.02, 95% CI 0.91-1.13; *P*=.77) with no heterogeneity in the estimate (*I*^2^=0%).

Four studies [26,28,30,31] reported attrition at 12-month follow-up that compared a tailored active intervention with an untailored passive control. The pooled effect of these 4 studies found a lower risk of attrition among the tailored intervention (RR=0.95, 95% CI 0.89-1.00; *P*=.07). This estimate was not statistically significant and had moderate heterogeneity (*I*^2^=36%). The test for subgroup differences found no significant differences between the active and passive subgroups at 12-month follow-up (χ^2^_1_=1.3; *P*=.25).

**Figure 6:**
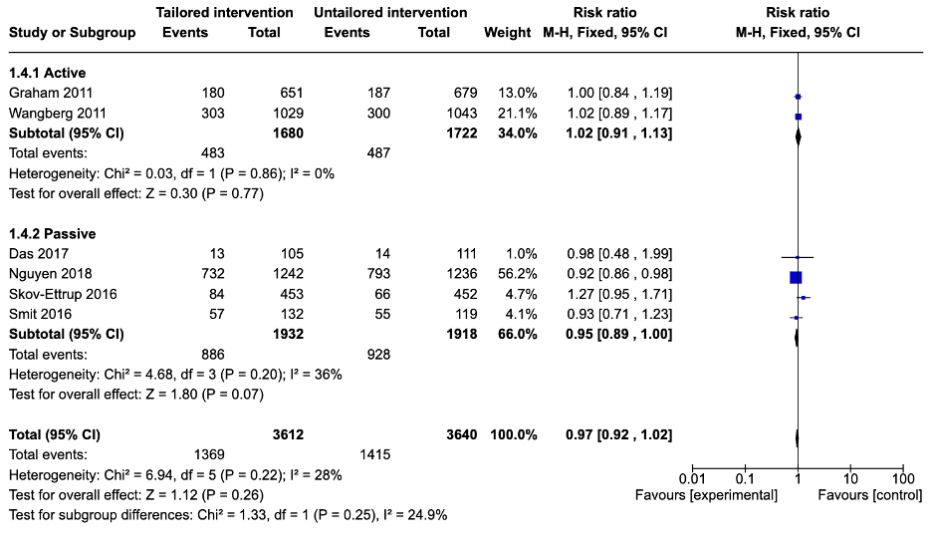
Comparison of attrition between tailored and untailored conditions at 12-month follow-up by control type.

At 12-month follow-up, the pooled effect of the 6 studies [21,23,26,28,30,31] (Figure 6) demonstrated a lower risk of attrition for the tailored intervention compared with the untailored control (RR=0.97, 95% CI 0.92-1.02; *P*=.26). This estimate was not statistically significant and had moderate heterogeneity (*I*^2^=28%), suggesting that tailoring had no effect on dropout attrition at 12 months.

### Sensitivity Analysis

Given the high heterogeneity of the results found for attrition at 1-, 3-, and 6-month follow-ups, a post hoc sensitivity analysis was conducted. Through visual inspection of the forest plots, the study by Westmaas [33] was suspected to be an outlier, and thus, this study was removed for the sensitivity analysis. Without the study by Westmaas [33] included in the meta-analysis, tailoring was found to have a higher risk of dropout attrition at 1-month follow-up (RR=1.03, 95% CI 0.96-1.11; *P*=.38) with high heterogeneity (*I*^2^=86%). Tailoring was found to have a lower risk of attrition both at 3-month follow-up (RR=0.99, 95% CI 0.94-1.03*; P*=.62) with high heterogeneity (*I*^2^=78%) and at 6-month follow-up (RR=0.99, 95% CI 0.94-1.05; *P*=.78) with moderate heterogeneity (*I*^2^=49%, Multimedia Appendix 4). As none of these estimates were statistically significant, the sensitivity analysis suggests that tailoring did not have a statistically significant effect on attrition at 1-, 3-, and 6-month follow-ups.

## Discussion

### Principal Findings

This study sought to understand the impact of tailoring web-based interventions on attrition among studies using active and passive control groups. Although several reviews have investigated the efficacy of web-based smoking cessation interventions [13-15,36], to our knowledge, this is the first systematic review that compared the rate of attrition of tailored and untailored web-based smoking cessation interventions. Our review found no differences in the likelihood of attrition between tailored web-based interventions and untailored controls at 1, 3, 6, or 12-month follow-ups.

The results of this systematic review align with the findings of previous studies. Strecher [37] conducted a path analysis of tailoring in web-based smoking cessation interventions and found that the relationship between tailoring depth and smoking cessation was weakly mediated by longitudinal engagement. Moreover, in a trial of a web-based smoking cessation program, the number of web pages opened (a measure of engagement) did not predict 12-week cessation [38]. Thus, the results of this review, as well as previous studies, suggest that retention may not mediate the impact of tailoring on smoking cessation in the long term.

When considering the engagement level (active vs passive) of the control intervention, tailoring was found to have no effect early on at 1-month follow-up but increased attrition by the 3-month follow-up for studies with an active control. This increase in attrition, however, was diminished at 12-month follow-up. These results suggest that for studies with an active control group, tailoring had no effect on attrition in the long term at 12-month follow-up. The opposite was found to be true among studies that compared a tailored active intervention with an untailored passive control group, where tailoring was associated with increased attrition at 1-month follow-up and decreased attrition at 3-month follow-up. This effect diminished over time, with no effect of tailoring on attrition at 12-month follow-up. When stratified by the type of control intervention, significant differences in the effect of tailoring were found between the active and passive subgroups at 1-, 3-, and 6-month follow-ups.

A potential explanation for the increased attrition for studies with an active control is that participants in the active tailored intervention group may derive benefits from the intervention early on and, thus, seize their participation before the end of the study. For studies with a passive untailored control, the opposite effect may have been observed, as there may be fewer other components to maintain interest in the intervention and in the study. Additional research is needed, however, to investigate this effect.

An important finding of this review was that only a few studies used an active control group (n=4), which suggests a lack of RCTs that compare web-based interventions with active control groups. The lack of active control groups may stem from the application of traditional RCT designs, well suited for investigating drug efficacy, onto digital health interventions [39]. Although robust methods such as the RCT are important, web-based interventions often have multiple active ingredients, including content and technology features. As such, there is a need to consider what the active ingredients of the technology are hypothesized to be to isolate them between the intervention and the control.

Another notable result was that all studies included in the review took place in the Western countries—a finding that has been reported by a previous review [36]. Given that developing countries continue to be targeted by tobacco companies [40], face difficulties in adopting tobacco strategies [41], and are projected to account for 80% of smoking-related deaths in the next century [42], studies are needed to evaluate the effectiveness of web-based smoking cessation interventions in populations outside of the Western countries. Tailoring interventions for these populations may require consideration of different variables (ie, cultural factors) and have unique impacts on attrition and effectiveness.

### Implications for Future Work

The findings of this review highlight the importance of selecting an appropriate control condition when evaluating the effectiveness of web-based smoking cessation interventions and identifying the important design components of these interventions. Where possible, web-based interventions should be compared with interventions that require the same amount of engagement to isolate the hypothesized active ingredients of the intervention. Without appropriate control interventions, studies may risk misrepresenting the benefits and mechanisms of intervention features, such as tailoring.

In this study, tailoring was intentionally operationalized as a unitary construct [43] to investigate the impact of tailoring on attrition. Although beyond the scope of this review, future research is needed to explore more specific questions related to tailoring, such as the impact of various tailoring strategies on the effectiveness of web-based smoking cessation interventions. The findings of this review suggest, however, that an investigation of specific tailoring strategies may be difficult because of unclear reporting of tailoring among the included studies. Future studies on tailored web-based smoking cessation interventions should more clearly outline the tailored components using the tailoring reporting standards proposed by Harrington [44]. Reporting in accordance with these standards will facilitate more nuanced analyses of the differential impact of various tailoring strategies.

### Limitations

The findings of this review should be interpreted with caution because of several limitations. First, multiple studies included in the review were found to be of unclear or high risk of performance bias. Second, the high heterogeneity in the intervention type and components, as well as the study design, made it difficult to pool the data on attrition and generalize the results of the study. Indeed, some studies included in this review had interventions that were both tailored and interactive [21-24,27,32]. This is an important limitation of this study, as interactivity can include personalization but not in all cases. Although previous reviews have grouped tailoring and interactivity [14], we sought to isolate the impact of tailoring and, thus, did not include interactivity. Some studies also had features in addition to tailoring and interactivity, such as social support or coaching, which could have impacted attrition. Third, in this review, we focused on dropout attrition and thus our findings may not capture the impact of tailoring on nonusage attrition. As only 1 study included in this review reported nonusage attrition, we anticipate that such analyses may be difficult to perform without improvements in reporting. Fourth, this review did not account for user acceptance and experience of technologies, which may have affected attrition, particularly in the short term. Finally, given that technologies are rapidly being developed both for research and in the consumer market, this review may have missed web-based interventions that were publicly available, especially outside of the Western countries. The restriction on published RCT studies in the English language may have contributed to the lack of interventions delivered internationally.

### Conclusions

This study aimed to investigate the impact of tailoring on attrition in web-based smoking cessation interventions. A systematic review of the literature yielded 14 RCTs that compared tailored web-based interventions with untailored control interventions, with 4 (29%) of the studies using an active control intervention and 10 (71%) studies using a passive control intervention. A meta-analysis of attrition reported by 86% (12/14) of the included studies found no effects of tailoring on attrition at 1, 3, 6, and 12-month follow-ups. The findings of this review suggest that tailoring may not be associated with reduced rates of attrition in web-based smoking cessation studies in the long term, although the high heterogeneity of effects indicates that the findings should be interpreted with caution. Future studies that incorporate active web-based controls, compare the impact of different tailoring strategies, and include populations outside of the Western countries are needed. Moreover, although beyond the scope of this review, future reviews may consider the impact of other technology features on attrition. It is with a greater understanding of how intervention features impact attrition that technologies may be better designed to retain users who may benefit from web-based smoking cessation interventions.

## Appendix

Multimedia Appendix 1 PRISMA (Preferred Reporting Items for Systematic reviews and Meta-Analyses) checklist.

Multimedia Appendix 2 Sample search strategy (MEDLINE: Medical Literature Analysis and Retrieval System Online).

Multimedia Appendix 3 Characteristics of the included studies.

Multimedia Appendix 4 Sensitivity analysis of tailoring at 1-, 3-, and 6-month follow-ups.

## Abbreviations

MEDLINE: Medical Literature Analysis and Retrieval System Online
PRISMA: Preferred Reporting Items for Systematic reviews and Meta-Analyses
RCT: randomized controlled trial
RR: risk ratio
