# Supplementary material for: Tailored Web-Based Smoking Interventions and Reduced Attrition: Systematic Review and Meta-Analysis
Source: J Med Internet Res. 2020 Oct 19;22(10):e16255. doi: 10.2196/16255 (PMC7605982; doi:10.2196/16255)
Supplement: Multimedia Appendix 2 [file jmir_v22i10e16255_app2.pdf]

Multimedia Appendix 2. Search Strategy (MEDLINE)

**Name of the database**	**MEDLINE**
**Platform**	**OVID**
**Database Coverage**	**1946 to Present (May 15, 2018)**
**Date exported to Reference Management Software (EndNote)**	
**Search Strategy**	
1	(internet* based or web* based or computer-assisted or www or ICT or Internet or website or eHealth or internet support or automated or internet* delivered or online* or web* page or web*application or internet*mediated or internet*supported or medical informatics or information tech* or e*health or e*therap* or e*mental health or emental health).mp. [mp=title, abstract, original title, name of substance word, subject heading word, keyword heading word, protocol supplementary concept word, rare disease supplementary concept word, unique identifier, synonyms]
2	(tailor* or personalized or customized or computer-tailored).mp. [mp=title, abstract, original title, name of substance word, subject heading word, keyword heading word, protocol supplementary concept word, rare disease supplementary concept word, unique identifier, synonyms]
3	(smok* cessation or smok* cessation intervention or quit smok* or stop smok*).mp. [mp=title, abstract, original title, name of substance word, subject heading word, keyword heading word, protocol supplementary concept word, rare disease supplementary concept word, unique identifier, synonyms]
4	(randomi* contro* trial or rct or randomi* contro* trial or clinical trial or controlled clinical trial).mp. [mp=title, abstract, original title, name of substance word, subject heading word, keyword heading word, protocol supplementary concept word, rare disease supplementary concept word, unique identifier, synonyms]
5	1 and 2 and 3 and 4
6	limit 5 to english language
