# Supplementary material for: Tailored Web-Based Smoking Interventions and Reduced Attrition: Systematic Review and Meta-Analysis
Source: J Med Internet Res. 2020 Oct 19;22(10):e16255. doi: 10.2196/16255 (PMC7605982; doi:10.2196/16255)
Supplement: Multimedia Appendix 3 [file jmir_v22i10e16255_app3.docx]

|  |  |  |  |  |  |  |
| --- | --- | --- | --- | --- | --- | --- |
| Multimedia Appendix 3. Characteristics of the included studies. | | | | | | |
| *Author, year* | ***Setting, Sample & Population*** | ***Intervention*** | ***Theoretical Framework Used*** | ***Tailoring Features*** | ***Control*** | ***Outcome*** |
| An,  2008 | USA  (n=517)  72.9% F  19.9 Age | *Real U*: Online college life magazine to (1) report health and lifestyle habits (smoking, drinking, stress); (2) take quiz with tailored educational feedback; and (3) read articles. Weekly personalized e-mails from peer coaches. | Social Cognitive Theory and Problem Behaviour Theory | Online, interactive quiz with tailored educational feedback on a smoking-related or general topic.  Personalized weekly e-mails based on information entered in website. | E-mail with links to online health (smoking cessation website, student health services) and academic resources (student academic services website). | Personalized smoking messages increased rates self-reported 30-day abstinence, but not 6-months sustained abstinence, measured at 6 months follow-up. |
| Borland, 2013 | Australia  (n=3530)^a^  60% F  42.1 Age | *QuitCoach*: Automated, program generating two- to four- page letters containing personalized cessation advice, strategies, and encouragement in addition to untailored resources. | Cognitive Behavioural Therapy | Advice in letters is based on answers to an assessment questionnaire; advice changes with progress in quit attempts. | [Web-](http://www.quitnow.org.au/) and telephone-based (quitline number) resources available in Australia. | No significant differences in self-reported 6-months sustained abstinence between the control and any intervention arm. |
| Das,  2017 | USA  (n=216)  34% F  39 Age | (1) Tailored computer-assisted smoking cessation advice reports printed for patients and providers; (2) nicotine replacement therapy (NRT); (3) optional NRT patch; (4) 15-30min counselling for motivational and decisional assistance. | Transtheoretical Model | Reports tailored (feedback and content matching) to transtheoretical model stage of change.  Computer intervention stored previous responses and provided feedback on progress and recommendations to quit and maintain abstinence (ipsative feedback). | *Usual care*: In-hospital NRT, smoking cessation pamphlet, and brief advice) | Significant differences in self-reported 7-day point prevalence tobacco abstinence (validated by breath carbon monoxide monitor) between intervention and control at 12-months follow-up. |
| Graham, 2011 | USA  (n=2005)  51.1% F  35.9 Age | [*QuitNet*](http://quitnet.com/): Interactive, individually tailored commercial website with information based on national guidelines (advice to quit, setting a quit date, problem-solving and skill-training) and online social support. | - | Individually tailored information and assistance on using approved pharmacotherapies based on assessment of demographics, motivation, smoking history, and nicotine dependence. | Free access to static website with the same information as QuitNet, without the tailored, interactive, social support features. | No significant differences in self-reported 30-day single point prevalence  abstinence between QuitNet and control at any timepoint (3-,6-,12-, or 18-months follow-up). |
| Harrington, 2016 | USA  (n=1488)  48% F  41.6 Age | [*Decide2Quit*](http://decide2quit.org/):  (1) Website with information on topics related to smoking cessation (making a quit plan, benefits of quitting, links to smoking cessation resources and interactive tools) and an extra webpage on the impact of smoking on health recovery.  (2) Asynchronous communication with Tobacco Treatment Counsellor.  (3) Semi-tailored automated e-mails with messages from peers and experts.  (4) Follow-up contacts with staff 7-14 days post-discharge to promote website use. | Social Cognitive Theory and Transtheoretical Model | Personalized asynchronous assistance from a Tobacco Treatment Counsellor.  Semi-tailored automated e-mails based on readiness to quit and health area of interest (e.g. cancer, wound healing, cardiovascular, respiratory, or general health) | *Usual care:* Brief advice by hospital staff, quit plan template handout with resources (e.g. quitline), brief advice in hospital admission handbook and discharge paperwork). | No significant differences in self-reported 30-day abstinence between the web-based intervention and control at 6-months follow-up. |
| Mavrot,  2016 | Switzerland  (n=1160)^a^  65.7% F  36.5 Age | *Coach:* Access to StopTobac website in addition to (1) automated personalized reports; (2) personal webpage with graph of individuals progress over time (e.g. motivation, self-efficacy, withdrawal symptoms); (3) series of automatic, tailored e-mails. | Transtheoretical Model | Automated personalized reports with 30 feedback items (e.g. text, graphs, images). Personalization based on responses to questionnaire  E-mails tailored to the individual’s smoking status, quit date, and level of dependence. | [*Stop-Tabac*](https://www.stop-tabac.ch/fr/)*:* Free website with information (e.g. text, graphics, videos, forums) about tobacco addiction and quitting (e.g. relapse prevention, quitting tips). | No significant differences in self-reported 1-month abstinence at 3-months follow-up. |
| Nyguyen, 2018 | France  (n=2478)  64.8% F  35.9 Age | [*E-coaching*](http://www.tabac-info-service.fr/): Fully automated program delivering 45 e-mails over the course of 3.5 months. | Transtheoretical Model, Motivational Interviewing, and Cognitive Behavioural Therapy | Registrants segmented into the 14 profiles (e.g. age, sex, level of dependence, previous quit attempts, pregnant women) based on responses to baseline questionnaire. Information appropriate to each profile was added to e-mails (e.g. Pregnancy of Tobacco for pregnant women)  E-mail content (e.g. advice to quit prior to quit date, congratulatory on quit date) and frequency (frequency of e-mails high initially and decreases over time, ending at 3 months after quit date) tailored to quit date. | *J’arrête de fumer:* 30-page PDF of a booklet available free online. Booklet structure is based on the Transtheoretical Model, with each chapter containing information, advice, and exercises pertaining to the smoker’s stage of change. | Significant differences in self-reported 7-day abstinence between e-coaching and control at 3-months follow-up (end of intervention), but not at 6- or 12-months follow-up. |
| Pardavila-Belio, 2015 | Spain  (n=255)  61.9% F  20.2Age | Multicomponent intervention consisting of: (1) 50 minutes of motivational interviewing by nurse; (2) [online self-help website](https://teen.smokefree.gov/); and (3) e-mail support and group therapy. | Theory of Triadic Influence | Online self-help website was tailored for young adult population. Topics included decisions, moods, effects of smoking, social life, quitting. | Brief advice and self-help pamphlet called “Stop smoking”. | Significant differences in self-reported 7-day abstinence (with verification using urine cotinine analysis) at 6-months follow-up. |
| Skov-Ettrup, 2016 | Denmark  (n=1810)^a^  57-58%F  52 (E-quit)  53 (Control) | [*E-quit*](http://ekvit.dk/): Website containing: (1) individual page with overview of program and feedback based on quit date; (2) exercises (text-and image-based) to increase motivation and coping; (3) blog for users to create, read, or comment; (4) action plan tool to identify individual coping strategies; (5) urgent assistance for cravings; (6) library on smoking and health  Optional tailored e-mails and text messages. | Self-Regulation  Theory, Transtheoretical Model, Social  Cognitive Theory, and Appreciative Inquiry | Tailored feedback based on Fagerström Test for Nicotine Dependence.  Optional e-mails and text messages with content tailored to quit date, preferred coping strategies, and responses to Wisconsin Inventory of Smoking Dependence Motives (WISDM-68). Frequency of text messages were also tailored to the quit date, with the highest intensity of messages sent near the quit date. | *Self-help booklet*: 36-page booklet on identifying difficult situations, coping strategies, setting a quit date, test for nicotine dependence, and information about pharmacotherapies. | No significant differences between the e-quit intervention and control in self-reported prolonged abstinence for 12 months after the intervention. |
| Smit,  2016 | Netherlands  (n=414)  59.9% F  48 Age | E-mail delivered computer tailored feedback letters (4-5 pages in length) after questionnaire completion. Letters contain feedback on: the pros and cons of smoking and quitting; perceived social influence; perceived self-efficacy to quit; action plans; and coping plans. | I-Change Model | Feedback and personalized letters based on individual characteristics (gender, attitude, smoking behaviour, social influence, self-efficacy, and coping plans) entered into questionnaires. | *Usual care*:  According to Dutch standard practice, ranges from brief smoking cessation advice to counselling (4 or more sessions). | Computer-tailored program was no more effective than usual care on self-reported prolonged abstinence at 6- (4.5 months of abstinence) or 12-months (6 months of abstinence) follow-up. |
| Tsoh,  2010 | USA  (n=42)  100%F  27.1 Age | *Video Doctor*: Laptop-delivered web-based multimedia intervention displaying interactive messages by a video doctor (actor-portrayed). Each intervention session concludes with automatically printed documents (1) cueing sheet for providers and (2) educational worksheet for the patient. | Motivational Interviewing | Program uses patient input and branch logic to deliver tailored messages (video clips based on user’s risk profile. | *Usual care*: prenatal appointments as usual with behavioural counseling delivered at the discretion of the clinician. | No significant differences in 30-day abstinence rate at 2-months follow-up between the intervention and control. |
| Wangberg, 2011 | Norway  (n=2298)  72.2% F  37 Age | *Slutta.no*: Website consisting of (1) advice and information about smoking cessation; (2) interactive tests for nicotine dependence, level of motivation, and smoker type (i.e. stress, comfort); (3) asynchronous social interaction features (e.g. personal diary, discussion forum, guestbook, visiting profiles of other users). | Health Action Process  Approach | Personalization: Up to 150 tailored messages. Frequency of message higher initially (daily), with the frequency reducing by the first 3 months, and substantially reducing 3 months after the quit date. | Multicomponent web-based intervention without tailored messages. | Higher 7-day abstinence rate for the intervention group at 1- and 3-months, but not at 12-months follow-up. |
| Westmaas, 2018 | USA  (n=1070)  83.3% F  40.3 Age | *Deluxe E-mail Group:* 27 tailored e-mails containing social support, motivational messages, and information on quitting (from Break  Away from the Pack series). E-mails were sent over the course of 2 months.  Certificate of abstinence e-mail automatically sent to participants 4 weeks after quit date. | - | Number, frequency, and timing of e-mails tailored to quit date (daily during the first week after the quit date, every other day the second week, every 2 days for the third week, and weekly for the next 7 weeks).  Tailoring of two e-mails based on responses to baseline questionnaire: (1) e-mail with information on NRT and other pharmacotherapies if participants reported that they planned to use NRT and (2) e-mail with coping strategies based on top 5 triggers identified at baseline. | *Standard E-mail Group:* a single untailored e-mail containing links to smoking cessation resources  ([ACS’s](http://www.cancer.org/%20%20Healthy/StayAwayfromTobacco/GuidetoQuittingSmoking/%20%20index)  [Guide to Quitting Smoking](http://www.cancer.org/%20%20Healthy/StayAwayfromTobacco/GuidetoQuittingSmoking/%20%20index) and [ACS’s Quitting Smoking—](http://www.cancer.org/Healthy/StayAwayfromTobacco/quitting-smoking-help-for-cravings-and-tough-situations)  [Help for Cravings and Tough Situations](http://www.cancer.org/Healthy/StayAwayfromTobacco/quitting-smoking-help-for-cravings-and-tough-situations)). E-mail was sent the same day, or day after, completing the baseline questionnaire. | Significant increases in 7-day point prevalence abstinence at 1-, 3-, and 6-months follow-up. |

^a^ Reported total randomized sample from a multi-arm RCT
