# Supplementary material for: Tailored Web-Based Smoking Interventions and Reduced Attrition: Systematic Review and Meta-Analysis
Source: J Med Internet Res. 2020 Oct 19;22(10):e16255. doi: 10.2196/16255 (PMC7605982; doi:10.2196/16255)
Supplement: Multimedia Appendix 4 [file jmir_v22i10e16255_app4.pdf]

Multimedia Appendix 4. Sensitivity analysis of tailoring at 1-, 3-, and 6-month follow-ups

Study or Subgroup	Tailored intervention Events	Tailored intervention Total	Untailored intervention Events	Untailored intervention Total	Weight	Risk ratio M-H, Fixed, 95% CI
**2.1.1 Active**						
Wangberg 2011	613	1029	640	1043	90.1%	0.97 [0.91 , 1.04]
**Subtotal (95% CI)**		**1029**		**1043**	**90.1%**	**0.97 [0.91 , 1.04]**
Total events:	613		640			
Heterogeneity: Not applicable						
Test for overall effect: Z = 0.83 (P = 0.41)						
**2.1.2 Passive**						
Skov-Ettrup 2013	106	453	64	452	9.1%	1.65 [1.25 , 2.19]
Tsoh 2010	5	23	5	19	0.8%	0.83 [0.28 , 2.43]
**Subtotal (95% CI)**		**476**		**471**	**9.9%**	**1.59 [1.21 , 2.08]**
Total events:	111		69			
Heterogeneity: Chi² = 1.48, df = 1 (P = 0.22); I² = 33%						
Test for overall effect: Z = 3.33 (P = 0.0009)						
**Total (95% CI)**		**1505**		**1514**	**100.0%**	**1.03 [0.96 , 1.11]**
Total events:	724		709			
Heterogeneity: Chi² = 13.84, df = 2 (P = 0.0010); I² = 86%						
Test for overall effect: Z = 0.88 (P = 0.38)						
Test for subgroup differences: Chi² = 11.81, df = 1 (P = 0.0006), I² = 91.5%						

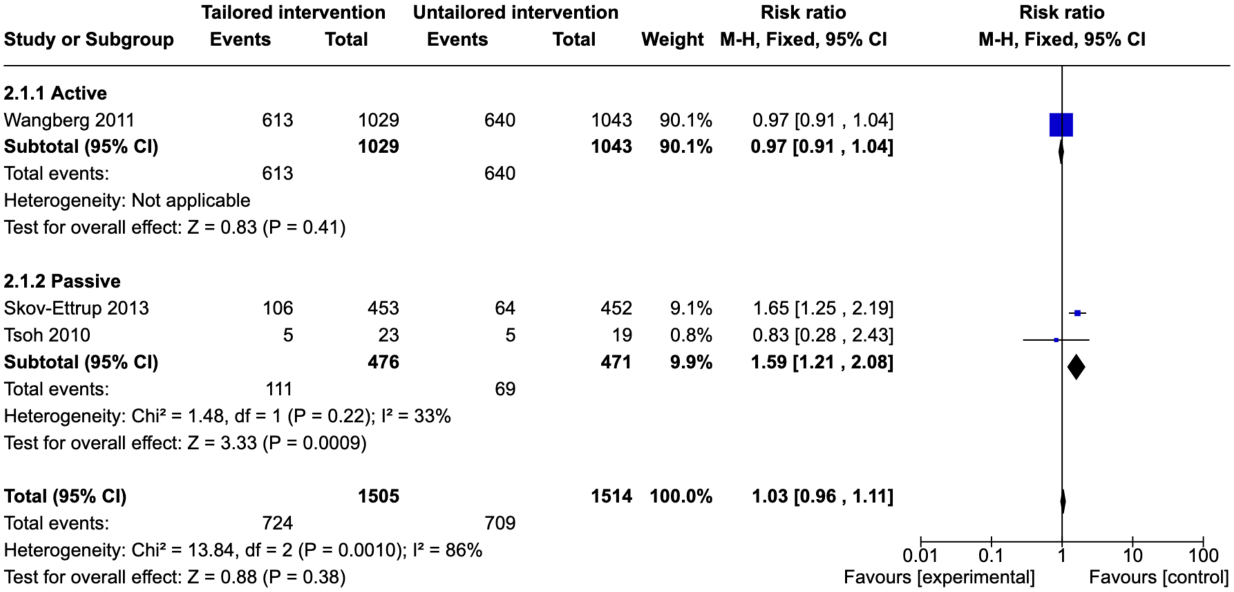

Sensitivity analysis at 1- month follow-up

Study or Subgroup	Tailored intervention Events	Tailored intervention Total	Untailored intervention Events	Untailored intervention Total	Weight	Risk ratio M-H, Fixed, 95% CI
**2.2.1 Active**						
Graham 2011	151	651	142	679	7.8%	1.11 [0.91 , 1.36]
Mavrot 2017	290	580	251	580	14.2%	1.16 [1.02 , 1.31]
Wangberg 2011	648	1029	644	1043	36.1%	1.02 [0.95 , 1.09]
**Subtotal (95% CI)**		**2260**		**2302**	**58.1%**	**1.06 [1.00 , 1.13]**
Total events:	1089		1037			
Heterogeneity: Chi² = 3.43, df = 2 (P = 0.18); I² = 42%						
Test for overall effect: Z = 2.11 (P = 0.03)						
**2.2.2 Passive**						
Das 2017	16	105	20	111	1.1%	0.85 [0.46 , 1.54]
Nguyen 2018	639	1242	720	1236	40.8%	0.88 [0.82 , 0.95]
**Subtotal (95% CI)**		**1347**		**1347**	**41.9%**	**0.88 [0.82 , 0.95]**
Total events:	655		740			
Heterogeneity: Chi² = 0.02, df = 1 (P = 0.89); I² = 0%						
Test for overall effect: Z = 3.43 (P = 0.0006)						
**Total (95% CI)**		**3607**		**3649**	**100.0%**	**0.99 [0.94 , 1.03]**
Total events:	1744		1777			
Heterogeneity: Chi² = 17.92, df = 4 (P = 0.001); I² = 78%						
Test for overall effect: Z = 0.50 (P = 0.62)						
Test for subgroup differences: Chi² = 15.94, df = 1 (P < 0.0001), I² = 93.7%						

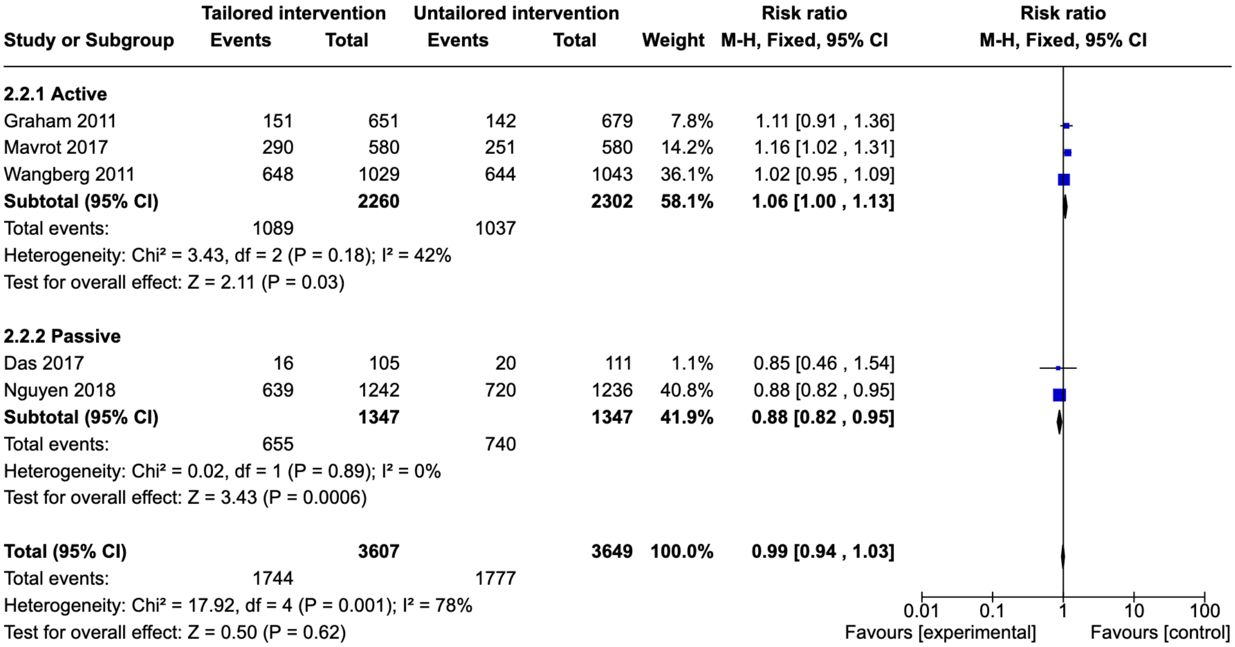

Sensitivity analysis at 3-months follow-up

Study or Subgroup	Tailored intervention Events	Tailored intervention Total	Untailored intervention Events	Untailored intervention Total	Weight	Risk ratio M-H, Fixed, 95% CI
**2.3.1 Active**						
Graham 2011	168	651	154	679	10.2%	1.14 [0.94 , 1.38]
Mavrot 2017	353	580	331	580	22.4%	1.07 [0.97 , 1.17]
**Subtotal (95% CI)**		**1231**		**1259**	**32.5%**	**1.09 [1.00 , 1.19]**
Total events:	521		485			
Heterogeneity: Chi² = 0.38, df = 1 (P = 0.54); I² = 0%						
Test for overall effect: Z = 1.87 (P = 0.06)						
**2.3.2 Passive**						
Das 2017	13	105	15	111	1.0%	0.92 [0.46 , 1.83]
Harrington 2016	98	748	89	740	6.0%	1.09 [0.83 , 1.42]
Nguyen 2018	667	1242	729	1236	49.3%	0.91 [0.85 , 0.98]
Pardavilaâ-Belio 2015	19	133	11	122	0.8%	1.58 [0.79 , 3.19]
Skov-Ettrup 2016	67	453	75	452	5.1%	0.89 [0.66 , 1.21]
Smit 2016	89	132	74	119	5.3%	1.08 [0.90 , 1.30]
**Subtotal (95% CI)**		**2813**		**2780**	**67.5%**	**0.95 [0.89 , 1.01]**
Total events:	953		993			
Heterogeneity: Chi² = 6.59, df = 5 (P = 0.25); I² = 24%						
Test for overall effect: Z = 1.69 (P = 0.09)						
**Total (95% CI)**		**4044**		**4039**	**100.0%**	**0.99 [0.94 , 1.05]**
Total events:	1474		1478			
Heterogeneity: Chi² = 13.65, df = 7 (P = 0.06); I² = 49%						
Test for overall effect: Z = 0.27 (P = 0.78)						
Test for subgroup differences: Chi² = 6.25, df = 1 (P = 0.01), I² = 84.0%						

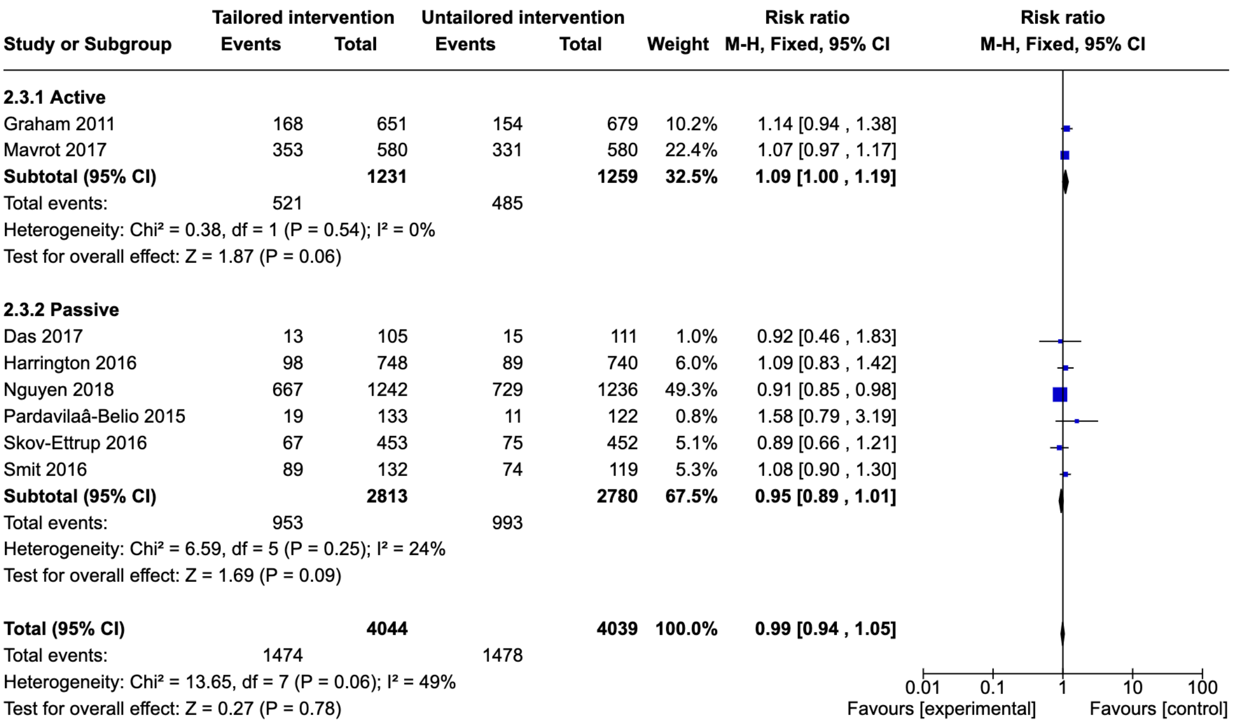

Sensitivity analysis at 6-months follow-up
